# Group Intensive Cognitive Activation in Patients with Major or Mild Neurocognitive Disorder

**DOI:** 10.3389/fnbeh.2016.00034

**Published:** 2016-02-29

**Authors:** Simonetta Panerai, Domenica Tasca, Sabrina Musso, Valentina Catania, Federica Ruggeri, Alberto Raggi, Stefano Muratore, Giuseppina Prestianni, Cinzia Bonforte, Raffaele Ferri

**Affiliations:** ^1^Unit of Psychology I. C., Oasi Institute for Research on Mental Retardation and Brain Aging (IRCCS)Troina, Italy; ^2^Unit of Neurology, Morgagni-Pierantoni HospitalForlì, Italy; ^3^Unit of Neurology I. C., Oasi Institute for Research on Mental Retardation and Brain Aging (IRCCS)Troina, Italy

**Keywords:** cognitive stimulation, cognitive training, dementia, mild cognitive impairment, patient-focused cognitive intervention

## Abstract

**Background**: No standard protocols are available for cognitive rehabilitation (CR) in conditions like Major or Mild Neurocognitive disorder (M-NCD or m-NCD, respectively); however, preliminary data seem to indicate that such interventions might have cost-effective beneficial effects and are free from side effect or adverse events. Three basic approaches are known: cognitive stimulation (CS), cognitive training (CT), and CR.

**Objective**: Aim of this study was to assess the efficacy of a protocol of group intensive cognitive activation (g-ICA) in patients with both M-NCD and m-NCD; the protocol was specifically arranged in our Research Institute, based on the principles of the central role of the patient and the mediation pedagogy.

**Subjects and Methods**: Sixteen patients with M-NCD and fifteen patients with m-NCD were enrolled, as well as eleven patients with M-NCD who were used as a control group (CG). The intervention was carried-out by a clinical neuropsychologist with daily group sessions over a period of 2 months. Neuropsychological assessment was performed at baseline and after the completion of the rehabilitative intervention.

**Results**: General cognitive functioning, attention, ideomotor praxis and visual memory scores were found to be significantly increased in all patients. Beneficial and significant effects were also found for constructive praxis in M-NCD and for executive functioning in m-NCD. All areas of the language function were significantly ameliorated in m-NCD, while this happened only for verbal repetition and syntax-grammar comprehension in M-NCD. No changes were detected for long- and short-term verbal memory, which were found to be worsened in controls without activation.

**Conclusion**: Our findings seem to indicate that g-ICA might be effective in inducing beneficial changes on the general cognitive functioning and other specific functions in patients with both m-NCD and M-NCD. Moreover, the specific protocol proposed, even if susceptible of important improvement, is easy to carry out within hospital facilities and cost-effective.

## Introduction

The Statistical Manual of Mental Disorders—fifth edition (DSM-5; American Psychiatric Association, 2013) provides the new cluster of neurocognitive disorders which includes three syndromes: delirium, mild, and major neurocognitive disorders (m-NCD and M-NCD respectively). M-NCD is largely synonymous with dementia, although the criteria have been modified so that impairments in memory or learning are not essential for the diagnosis, except for NCD due to Alzheimer’s disease (AD). M-NCD is characterized by the evidence of significant acquired deficits in one or more cognitive domains, based on concerns reported by the individual or the informants and preferably documented by standardized neuropsychological testing or quantified clinical assessment; such deficits must interfere with independence in everyday activities. m-NCD is synonym for mild cognitive impairment (MCI) and is characterized by a mild acquired cognitive decline not interfering with independence in everyday activities, even though greater effort or compensatory strategies may be required. The reason for diagnosing m-NCD resides in the increasing interest in early diagnosis and intervention to prevent or postpone dementia, although m-NCD is not always a precursor of dementia (Langa and Levine, 2014).

The DSM-5 also describes criteria for the diagnosis of specific etiological subtypes of both NCDM-NCD and m-NCD, based on their clinical features and biomarkers (e.g., Frontotemporal lobar degeneration, Traumatic Brain Injury, AD, Vascular Disease, Lewi body Disease, and Parkinson’s Disease), and consistent with those developed by various expert groups. Nevertheless, establishing the etiology in m-NCD is rather difficult and may remain unspecified in many patients. For some etiologies, the level of certainty is also well defined, with “probable” representing a higher level of certainty than “possible” (for a review of NCD, see Sachdev et al., 2014).

Options for treating M-NCD include both pharmacological and non-pharmacological therapies. At present, no drugs capable of modifying the disease and totally control the symptoms are available (Hildreth and Church, 2015); therefore, cognitive interventions have been gradually and increasingly focused as an emerging therapeutic approach which could aid prevention and treatment of dementia, especially combined with exercise and pharmacological therapy (Loewenstein et al., 2004; Law et al., 2014). The hypothesis that persons with neurocognitive diseases might benefit from cognition-based intervention derives from the concepts of neuronal plasticity and cognitive reserve and from a general view that the lack of cognitive activity hastens cognitive decline, in normal ageing individuals as well as those with dementia. However, according with some studies, deterioration cannot be stopped after 2 years of treatment in patients with dementia (Requena et al., 2006; Woods et al., 2012). Another study by Jedrziewski et al. (2014) carried out with community-resident and institutionalized persons, showed that engagement in cognitive activities was inversely associated with the onset of cognitive impairment at 5-year follow-up, but was no longer significant at 10-year follow-up, this resulting in the fact that cognitive interventions possibly lower the risk for cognitive impairments and dementia and might delay their onset.

Neural plasticity can be defined as the ability of the nervous system to adapt its structural organization in response to factors affecting its integrity and functioning. Protecting neural plasticity in brain regions typically affected in AD might improve the neurological functioning and prevent the loss of neuronal processes that occur in this disease (Buschert et al., 2010). Neural plasticity also plays a role in cognitive reserve which represents the brain’s ability to make flexible and efficient use of cognitive networks when performing tasks in the presence of brain pathology (Stern, 2002). Persons with higher cognitive reserve tend to have better clinical outcomes for any level of pathology and brain reserve (Stern, 2013). Among all the factors influencing cognitive reserve, mentally stimulating activities were shown to have the largest effect on the risk for dementia and might attenuate cognitive decline. Therefore, cognitive interventions could potentially delay the onset of dementia, as in the case of m-NCD, attenuate the clinical symptoms associated with M-NCD and partially reduce the speed of decline (Buschert et al., 2010). As compared with pharmacological treatment, cognitive intervention appears to be less expensive and more cost-effective (Buschert et al., 2010) and free from side effects or adverse events.

However, research in this area is rather scarce and evidence of effectiveness rather controversial (Alves et al., 2013); moreover, to date, no standard protocols are available for cognitive intervention in conditions such as M-NCD or m-NCD. Three main cognition-based approaches emerge from the literature: cognitive stimulation (CS), cognitive training (CT) and cognitive rehabilitation (CR; Clare and Woods, 2004; Buschert et al., 2010; Alves et al., 2013; Choi and Twamley, 2013).

CS includes a range of group activities with the aim to enhance general cognition and social functioning. This approach assumes that cognitive functions work together and should be stimulated at the same time in a social setting. CS is based on reality orientation (RO), first described (Taulbee and Folsom, 1966) as a technique to improve the quality of life of confused elderly people, although its origins lie in an attempt to rehabilitate severely disturbed war veterans. RO involves the presentation of orientation and memory information, relating, for example, to time, place, and person, in order to provide the person with a greater understanding of his/her surroundings, possibly resulting in an improved sense of control and self-esteem. The basic principles of RO have been incorporated in the everyday clinical practice and in new formats of treatments. Of notice, the cognitive stimulation therapy (CST), by Spector et al. (2003), a brief group intervention (14 person-centered sessions of themed enjoyable activities), designed for patients with dementia, has received evidence of its efficacy on cognitive functions and quality of life (Woods et al., 2012). CST seem to be independent of whether people are taking acetylcholinesterase inhibitor (AChEI) medication, and that being older or female implies increased cognitive benefits from the intervention; care home residents improve more than community residents on quality of life, but the community sample seems to improve more in relation to behavior problems. This method has been published as a manual (Spector et al., 2006) and is the only non-pharmacological intervention recommended by the National Institute for Health and Clinical Excellence (2007) for the treatment of cognitive symptoms in dementia. It also appears to be cost-effective (Knapp et al., 2006).

CT is an approach consisting of computer-based or paper-and-pencil cognitive exercises with different levels of difficulty, targeting specific cognitive functions (e.g., attention, memory, problem-solving). It is performed individually or in group, with the aim to enhance targeted functions and teach compensatory techniques, generalizable to daily life. There is evidence, in pre-dementia and mild-to-moderate AD, of cognitive improvements (Cipriani et al., 2006; Talassi et al., 2007), but not of generalization (see Alves et al., 2013). CT is especially employed with MCI patients, mainly focusing on episodic memory, but also on attention, executive functions, language, visual-spatial abilities and processing speed (Simon et al., 2012). Computerized CT did not show higher improvements when compared to non-computerized training. CT also includes restorative (such as errorless learning, spaced retrieval, errorful learning, reminiscence therapy) and compensatory strategy (such as visual imagery, method of loci, mind mapping, cueing, external memory aids), frequently applied simultaneously. The results from different studies are rather inconsistent with regard to the enhancement of memory domain and generalization to other cognitive functions (Simon et al., 2012). A recent meta-analysis of randomized controlled trials by Wang et al. (2014) found significant effects on global cognitive functions, and weak evidence of improvements in executive functions and delayed memory. Very briefly, CST appears to be more effective in patients with dementia (Yuill and Hollis, 2011), whereas CT seems to be more effective in patients with MCI (Buschert et al., 2010).

CR is an individualized approach, which employs any intervention strategies that might enable patients and their families to manage cognitive deficits, considered within the interaction between the patient and the environment. The main difference between this approach and the CT is the full involvement of the family in the treatment. Compensatory methods, verbal instructions and physical demonstrations are used (Choi and Twamley, 2013). To date, there are very few studies about this approach and evidence of CR benefits is still to be obtained (Alves et al., 2013).

Other non-pharmacological multicomponent approaches are described in the literature that substantially appear to be variations of CT, for example interventions combining CT with RO (Raggi et al., 2007), with physical exercises and CS (Olazarán et al., 2004), with activities of daily living (ADL; Avila et al., 2004), or with transcranial magnetic stimulation (Bentwich et al., 2011). Some authors (Yamaguchi et al., 2010; Yamagami et al., 2012), shifting from “what” is taken to “how” it is taken, suggested a combined treatment, that is the brain-activating rehabilitation (BAR), based on five principles: enjoyable activities in an accepting atmosphere, empathetic communication between patients as well as between the therapist and the patients, enhancing motivation by means of praises, conferring a social role to each patient (based on his/her remaining abilities), errorless learning in order to maintain the patient’s dignity; evidence of a decreased social withdrawal and global severity of dementia was provided by a randomized clinical trial but no difference was found in cognitive tests.

In summary, taken as a whole, previous studies show evidence for small but consistent effects of non-pharmacological cognitive interventions in improving global cognitive functions in patients with both MCI or dementia, especially AD. At present, however, no standardized intervention programs have been designed yet and research in this field “is still in its infancy, and in spite of the growing evidence of its effectiveness, is still lacking recognition among health professionals as well as caregivers” (Alves et al., 2013). Therefore, the search for additional evidence concerning the efficacy of these approaches for each type of dementia and deterioration stages is strongly recommended. Considering that the worldwide number of patients with dementia is expected to dramatically increase, the need to implement cognitive interventions in inpatient and outpatient services for subjects with NCD has become more and more compelling.

The aim of this article is to report preliminary results on the implementation of the group intensive cognitive activation (g-ICA), a combined treatment properly designed in our Research Institute, and delivered in an inpatient hospital setting to persons with M-NCD and m-NCD, on the basis of their cognitive outcomes. This approach represents a proposal aimed at the enrichment of the traditional rehabilitation programs provided in hospitals, generally based on physical and language therapy.

## Materials and Methods

### Participants

The patient group included 16 subjects (6 males and 10 females) with mild-to-moderate M-NCD and 15 patients with m-NCD (7 males and 8 females), diagnosed on the basis of the DSM-5 criteria by a multidisciplinary team (including a senior geriatrician, a neurologist, and a psychologist) before their admission at the Rehabilitation Unit of our Institute. As far as the M-NCD sample is concerned, in seven patients the disease was due to possible AD, in three to vascular disease, in three to frontotemporal lobar degeneration-language variant, in one to frontotemporal lobar degeneration-behavioral variant, and in one to traumatic brain injury. Patients with M-NCD due to possible AD were treated with cholinesterase inhibitors soon after the completion of the diagnostic process. Within the m-NCD sample, in six patients the disease was due to possible AD, in five to vascular disease, in two to traumatic brain injury and in two to another medical condition (namely removal of benign brain tumors). Mean chronological age of patients with M-NCD was 64 years (±10.64 SD); early onset of the diseases (<65 years) was evident in 11 individuals. Mean chronological age of patients with m-NCD was 57 years (±9.82 SD). Patients with M-NCD were assigned to treatment groups on the basis of the severity of the disorder (mild or moderate) following the waiting list; patients with m-NCD were assigned to treatment groups following the waiting list.

Inclusion criteria to be met for M-NCD were as follows: (a) DSM-5 diagnostic criteria for M-NCD; (b) score between 10 and 23 at the mini mental state examination (MMSE; Folstein et al., 1975); (c) score between 40 and 85 (mild or moderate cognitive decline) at the milan overall dementia assessment (MODA; Brazzelli et al., 1994); (d) score between 1 and 2 at the clinical dementia rating (CDR; Hughes et al., 1982); (e) loss of almost one ADL (Katz et al., 1970) and/or instrumental activities of daily living (IADL; Lawton and Brody, 1969); and (f) patients presented with some communication abilities, no major sensorial (sight and hearing) or physical illnesses and no behavioral problems impairing their participation to group activities. Inclusion criteria to be met for m-NCD were as follows: (a) DSM-5 diagnostic criteria for m-NCD; (b) score between 24 and 26 at the MMSE; (c) score between 85.5 and 89 (borderline cognitive level) at the MODA; (d) score 0.5 at CDR; (e) ADL and IADL globally maintained; and (f) patients had no major sensorial (sight and hearing) or physical illnesses impairing their participation to group activities.

A control group (CG) was also recruited which included 11 elderly with M-NCD (2 males and 9 females) who received daily care and assistance, with daily animation activities and weekly attendance of religious events. Their mean age was 69 years (±7.87 SD). Inclusion criteria were: (a) score between 10 and 23 at the MMSE (Folstein et al., 1975); (b) score between 40 and 85 (mild or moderate cognitive decline) at the MODA (Brazzelli et al., 1994); (c) score between 1 and 2 at the CDR (Hughes et al., 1982); and (d) loss of almost one ADL (Katz et al., 1970) and/or IADL (Lawton and Brody, 1969).

### Procedures

g-ICA is an intensive combined group treatment including 30 cognitive activation sessions, delivered by a trained clinical neuropsychologist, supported by a practicing psychologist. A pre/post neuropsychological assessment was administered to all groups of patients.

Also for CG, two neuropsychological battery administrations were carried out, the last after 2 months from the first. CG did not benefit from the g-ICA program.

The neuropsychological battery and the arrangement of sessions and contents are described in the following paragraph.

### Neuropsychological Instruments

In order to overcome the weaknesses typically deriving from only one or two measures of the global cognitive functioning and with the aim of obtaining a wide-spectrum neuropsychological profile, a comprehensive neuropsychological battery was used, as suggested by Bianchi and Dai Prà (2008). Global cognitive functioning was evaluated by means of a number of measures, and namely the MMSE (Folstein et al., 1975), the MODA (Brazzelli et al., 1994), and the montreal cognitive assessment (MoCA, Nasreddine et al., 2005); to assess reasoning ability and intellectual level, the colored progressives matrices (CPM; Basso et al., 1987) were used; short term memory was assessed by means of the Digit Span (Orsini et al., 1987), the Serial repetition of two-syllable words (Spinnler and Tognoni, 1987), and test of Corsi (Orsini et al., 1987); verbal episodic memory (immediate and delayed recall) was investigated by means of the Rey’s 15 words (Carlesimo et al., 1996); non-verbal episodic memory by the Enhanced Cued Recall (Grober et al., 1988) and the Rey’s Complex Figure-memory reproduction (Carlesimo et al., 2002); selective attention was evaluated using the Digit Cancellation test (Spinnler and Tognoni, 1987); for the spatial cognition and constructional apraxia the copy of Rey’s Complex Figure (Caffarra et al., 2002) was used; ideomotor praxis was assessed by means of the Imitating Gestures Test (De Renzi et al., 1980); for the assessment of the language abilities, the Aachener Aphasia Test (Italian version) was employed (Luzzatti et al., 1996) and for the frontal functions, the frontal assessment battery (FAB; Dubois et al., 2000).

### The g-ICA Protocol

The g-ICA protocol is based on two general principles: the central role of the patient and the mediation pedagogy. The patient-centered principle consists in taking into account the patients’ needs and expectations. Patients with dementia are commonly willing to improve their health condition, maintain their cognitive and daily-living abilities while minimizing any memory loss; moreover, they need to be listened to, and receive emotional support (Bossen et al., 2009). Consequently, g-ICA was properly designed to include activities tapping on a wide spectrum of cognitive abilities, which appeal to the cognitive strength of patients, bringing them out of a vicious circle of failure toward a new virtuous circle based on capabilities and abilities. The principle of the mediation pedagogy comes from the theory of Structural Cognitive Modifiability which is, in practice, the mediated learning experience (Feuerstein, 1999). In our approach, mediation is focused on interpersonal relationships, characterized by a loop of dynamic and positive feedbacks between patients and the mediator; this latter, in a respectful and pleasant atmosphere, essentially encourages the patients—never standing in for them—to engage with stimuli and provides the required prompts, so they can actively and successfully complete the task. The mediator is required to show empathy, sense of humor and the ability to make cognitive activities as pleasant as possible. A package of proactive (antecedent) and reactive (consequent) procedures (Cooper et al., 2007) is used, in order to raise the patients’ motivation in the working area and during cognitive activities, and maintain self-esteem; namely: a prosthetic physical environment, made of a separate and comfortable room, equipped with a calendar, a wall clock and other visual cues, a computer, a video projector and a whiteboard; a 20 min break with a snack; errorless learning (prompts, prompt-fading and delayed prompt); variation of activities and materials; variation of task difficulties; use of social positive reinforcements; correction of errors, by highlighting positive aspects of the response and adding new stimuli aimed to lead patients to independently overview the global quality of their response.

The g-ICA is an intensive group treatment combining CS (including RO) and CT; in our setting, each group was made of four/five participants. Interventions were delivered in an inpatient hospital setting, with daily group sessions, each lasting approximately 3.5 h, 5 days a week (from Monday to Friday), over a period of 2 months. During the first and the last weeks, neuropsychological assessments were administered; cognitive activation sessions extended over a period of 6 weeks. Each session begun with the mediator welcoming the patients, then engaging in a little discussion about gossip/crime news recently happened or about facts that had taken place where patients resided; then, a short overview of the activities carried out in the last meeting was discussed and a RO activity (day, month, year, season, weather, time, name of participants) was started, followed by cognitive or non-cognitive tasks, a short break, and cognitive or non-cognitive tasks once again; finally, the overall course of the session and the level of engagement and feelings of patients were discussed. Contents were organized on a weekly basis, in order to stimulate a wide range of cognitive functions; the level of difficulty of the activities was adapted to the group cognitive capability (Table 1). Both paper-pencil and computer activities were employed to train specific cognitive functions.

**Table 1:** **Weekly plan of activities in g-ICA**.

Day	Cognitive functions stimulated	Activities examples for participants with M-NCD	Activities examples for participants with m-NCD
Every day	Global	Reality orientation (day, month, year, season, weather, time estimate, name of participants, building one’s own identity card and other personal information, temporal and spatial relations)	Reality orientation (date; mental calculations-hours and days within a month, a season or a year)
	Ecological memory	Memory in the boxes: group game with personal objects; this game was taken from Florenzano (1988) and then adapted	Memory in the boxes (group game with neutral objects)
Monday/Thursday	Ideomotor praxis	Each participant, in turn, moves a part of his/her body (right or left); the other participants have to imitate the movement (right and left, accordingly); variations can include different number of repetitions and imitation of two series of movements	Each participant, in turn, moves a part of his/her body (right or left); the other participants have to imitate the movement (right and left, accordingly); variations can include imitation of three or more series of movements forward and backward
Monday/Friday	Global	Getting pairs of cards (it is a group card game; each set contains paired cards on established themes—such as food, objects, clothes, etc.; each participant receives 6–8 cards and is required to reconstruct the highest number of pairs following certain rules)	Getting pairs of cards (in this case, cards have to be paired not only on the basis of their sameness, but also on the basis of logical matching; each player can receive up to 20 cards)
	Visual, auditory and spatial memory	Little computerized and non-computerized memory games for adults (shapes, cards, black and white figures, flags, faces, colors, numbers, letters, smiles, word retrieval, sentences repetition, location of objects, etc.)	Big computerized and non-computerized memory games for adults
Tuesday/Thursday	Auditory and visual selective attention	Computerized and non-computerized non-verbal cancelation tasks/sounds and words recognition (one or two meaningful target stimuli)	Computerized and non-computerized non-verbal cancelation tasks/sounds and words recognition (two or more meaningful or non-meaningful target stimuli)
	Global	Functional tasks (telling time, counting money, using a calendar, reading simple instructions, using the mobile phone, etc.)	Functional tasks (solving daily math problems, understanding medicine labels, writing a phone message, using the internet, etc.)
Tuesday/Friday	Verbal language	Naming (objects, pictures, features, functions, classes); pictures description; sentences repetition; reading, comprehension, reconstruction of a brief story through pictures and written sentences; creating a story II (guided group play)	Sentences repetition; verbal fluency; verbal inference; creating a story I (each participant adds a new sentence at a time, keeping a logic sequence of the story events); creating a story II (guided work play).
Wednesday	Constructional praxis	Drawing tasks (figure copy or completion ); easy tangrams; cubes or matches constructions	Tangrams; identify and outline given figures within a cloud of dots; differentiating (divide a whole into its parts) and integrating (join parts into a whole); computerized and non computerized stencil design (through superimposed parts).
	Semantic memory	Starting from a central topic, all related information were retrieved by using a fixed diagram including category, environment, features, functions and free associations (Celentano et al., 2002)	
	Ecological problem solving	Starting from a visual presentation of a everyday problem situation, participants are required to express what they think about the situation and what they would do (tasks adapted from Schwartz, 1990, 1993).	Starting from a verbal presentation of a everyday problem situation, participants are required to analyze the situation, hypothesize the antecedents and the purposes of the characters, and finally to express what they think about the situation and what they would do

### Statistical Analysis

We first obtained descriptive statistics for all parameters measured in this study which served to calculate the Cohen’s *d* value. Cohen’s *d* is defined as the difference between two means divided by the pooled standard deviation for those means. According to Cohen, 0.2 is indicative of a small effect, 0.5 of a medium and 0.8 of a large effect size.

Most variables analyzed in this study did not show a normal distribution; for this reason, non-parametric statistics were subsequently used. Thus, the results obtained from the pre- and post-treatment assessments in both groups of patients and in both M-NCD subgroups were compared by means of the Wilcoxon test for paired data sets. Significance level was set as *p* < 0.05. However, as for each parameter four different comparisons were performed, also the Bonferroni correction was applied and the corresponding corrected significance level was set at *p* < 0.0125.

The comparison between the results of the first and second neuropsychological battery administration in the CG was carried out by means of the Wilcoxon test for paired data sets.

The comparison between the M-NCD group and controls was carried out by means of the Mann-Whitney U test. The significance level was set as *p* < 0.05.

### Ethics Approval

This study was carried out in accordance with the Regulations of the Local Ethics Committee “Oasi Maria SS.” (CE17/06/2013OASI) abiding by the National Regulations for Ethics Committees. Written informed consent was obtained by all volunteering subjects before entering the study, in accordance with the Declaration of Helsinki. The population involved in this study, albeit vulnerable, were able to personally give their consent to participate to the study. In case of patients with dementia, caregivers have been also informed about objectives and procedures of the research study.

## Results

Table 2 reports descriptive statistics and effect size of the results obtained in all groups of patients. The statistical significance of the difference between the results obtained from pre- and post-treatment assessments are reported in Table 3; the findings in m-NCD and M-NCD will be described separately.

**Table 2:** **Descriptive statistics and effect size of the results obtained in all groups of patients**.

	M-NCD all (*n = 16)*			M-NCD due to possible AD (*n* = 7)			M-NCD not due to AD (*n* = 9)			m-NCD (*n* = 15)			CG (*n* = 11)		
	Mean	SD	Cohen’s *d*	Mean	SD	Cohen’s *d*	Mean	SD	Cohen’s *d*	Mean	SD	Cohen’s *d*	Mean	SD	Cohen’s *d*
**MODA**
Pre	67.55	10.07	0.66	74.74	4.23	1.68	61.25	9.51	0.66	81.71	6.82	1.18	76.93	8.22	0.47
Post	74.61	11.09		82.39	4.86		67.81	10.61		88.73	4.90		73.04	8.23	
**MMSE**
Pre	15.80	4.26	0.83	18.63	2.07	1.39	12.97	4.05	0.91	24.29	2.66	0.73	20.37	2.02	0.90
Post	19.87	5.43		22.12	2.88		17.89	6.51		26.02	2.06		18.22	2.72	
**MoCA**
Pre	11.78	5.17	0.55	14.40	2.97	0.97	8.50	5.80	0.43	19.5	5.58	0.96	Not administered		
Post	14.78	5.76		17.80	3.96		11.00	5.77		32.71	2.64		Not administered		
**Colored progressive matrices**
Pre	18.19	6.63	0.54	18.63	4.15	0.67	17.80	8.53	0.55	26.11	5.01	0.67	18.95	2.77	0.77
Post	21.74	4.98		21.77	5.21		21.70	5.15		29.13	3.95		16.68	3.08	
**Digit span**
Pre	3.80	1.35	0.32	4.68	0.98	0.76	3.03	1.17	0.11	3.83	1.18	0.26	4.59	0.38	0.56
Post	3.40	1.12		3.96	0.91		2.91	1.10		4.10	0.92		4.32	0.56	
**Corsi’s test**
Pre	3.38	1.22	0.11	3.07	0.97	0.30	3.66	1.42	0.33	3.43	1.27	0.29	4.32	0.76	0.12
Post	3.23	1.43		3.36	0.98		3.14	1.76		3.80	1.29		4.23	0.69	
**Serial repetition of two syllabic words**
Pre	2.55	0.79	0.21	2.96	0.51	0.52	2.19	0.84	0.10	2.73	0.86	0.71	3.66	0.39	0
Post	2.75	1.10		3.25	0.60		2.31	1.28		3.33	0.84		3.66	0.39	
**Rey’s 15 words-immediate recall**
Pre	22.94	8.35	0.11	26.63	8.28	0.10	19.71	7.42	0.26	34.82	5.75	0.58	28.99	6.47	0.67
Post	23.81	8.38		25.91	6.46		21.96	9.81		39.18	8.91		25.15	6.84	
**Rey’s 15 Words-delayed recall**
Pre	2.93	3.25	0.09	3.91	2.94	0.008	2.08	2.23	0.26	6.71	2.51	0.53	4.85	2.25	0.16
Post	2.66	2.31		3.89	2.14		2.69	2.44		8.19	3.03		4.47	2.59	
**Rey complex figure-memory reproduction**
Pre	6.78	6.82	0.21	2.20	1.68	0.96	12.50	6.45	0.37	13.75	5.69	0.40	4.32	3.47	0.16
Post	8.11	5.90		6.40	5.97		10.25	5.85		16.42	7.63		3.77	3.48	
**Enhanced cued recall**
Pre	7.60	4.07	0.27	7.57	2.51	0.41	7.63	5.26	0.54	13.64	2.9	0.10	14.73	1.01	0.66
Post	8.67	3.85		8.71	3.09		8.63	4.63		13.93	2.87		13.73	1.90	
**Digit cancellation test**
Pre	20.45	9.73	0.50	22.71	11.43	0.52	18.19	7.89	0.20	35.69	11.56	0.59	26.75	6.27	0.34
Post	25.60	10.95		27.82	8.11		23.66	11.84		41.98	9.62		24.45	7.14	
**Imitating gestures test**
Pre	41.04	11.21	0.81	42.50	13.16	0.92	39.57	9.69	0.56	59.40	7.52	1.02	46.41	6.31	0.94
Post	50.01	10.88		53.29	10.25		45.40	11.08		65.63	4.26		40.82	5.59	
**Rey complex figure-copy**
Pre	16.78	8.08	0.74	11.80	5.99	1.30	23.00	5.77	0.21	27.11	7.68	0.04	13.55	5.14	0.42
Post	21.94	5.75		20.30	7.03		24.00	3.49		27.43	9.68		11.41	5.08	
**Frontal assessment battery**
Pre	8.10	3.70	0.35	10.40	3.91	0.29	5.80	1.48	0.82	12.33	3.37	1.02	10.36	3.20	0.27
Post	9.30	3.20		11.40	2.88		7.20	1.92		15.14	1.96		9.45	3.45	
**AAT-Token test**
Pre	55.19	9.57	0.24	63.86	5.43	0.45	48.44	5.70	0.33	65.73	5.96	0.61	58.09	6.22	0.24
Post	57.56	10.52		66.71	7.11		50.44	6.27		68.93	4.46		56.45	7.61	
**AAT-Repetition**
Pre	56.47	9.16	0.33	64.00	7.21	0.15	49.88	4.09	0.72	63.47	5.80	0.12	51.36	4.27	0.28
Post	59.69	10.23		65.14	7.99		55.44	10.10		64.50	10.05		50.18	4.31	
**AAT-Written language**
Pre	57.88	9.27	0.15	63.86	8.90	0.28	53.22	6.78	0.08	68.27	5.82	0.32	54.36	4.72	0.12
Post	59.44	10.89		66.71	11.10		53.78	6.91		70.09	5.39		53.78	4.96	
**AAT-Naming**
Pre	55.94	10.16	0.40	63.00	8.29	0.60	50.44	8.02	0.40	75.13	14.95	0.03	55.55	6.49	0.17
Post	60.38	12.05		68.29	9.25		54.22	10.53		72.50	10.50		54.45	6.38	
**AAT-Comprehension**
Pre	52.63	7.37	0.23	57.00	7.59	0.29	49.22	5.36	0.23	64.79	8.29	0.27	51.55	4.95	0.12
Post	54.69	10.12		59.29	8.36		51.11	10.34		67.27	9.95		50.82	6.95	

**Table 3:** **Statistical significance of the differences between the results obtained from pre- and post-treatment evaluations in patients with m-NCD and M-NCD (Wilcoxon test)**.

	M-NCD all (*n* = 16) *p*	M-NCD due to possible AD (*n* = 7) *p*	M-NCD not due to AD (*n* = 9) *p*	m-NCD (*n* = 15) *p*
**Global cognitive function**
MODA	0.005*	0.018	0.008*	0.015
MMSE	0.008*	0.018	0.028	ns
MoCA	0.021	0.043	ns	0.008*
**Reasoning and cognitive level**
Colored progressive
matrices	ns	ns	ns	0.024
**Memory**
Digit span	ns	ns	ns	ns
Corsi’s test	ns	ns	ns	ns
Serial repetition of
two-syllabic words	ns	ns	ns	0.022
Rey’s 15 words-immediate recall	ns	ns	ns	ns
Rey’s 15 words-delayed recall	ns	ns	ns	ns
Rey’s complex figure-
memory reproduction	ns	ns	ns	0.007*
Enhanced cued recall	0.041	0.028	ns	ns
**Selective attention**
Digit cancellation test	0.048	ns	ns	0.008
**Praxis**
Imitating gestures test	0.002*	0.018	0.043	0.001*
Rey’s complex
figure-copy	0.044	0.043	ns	ns
**Frontal functions**
Frontal assessment battery	ns	ns	ns	0.002
**Language**
Aachener aphasia test
(AAT)—Token test	0.006*	0.028	ns	0.019
AAT—Repetition	0.009*	ns	0.028	ns
AAT—Written language	ns	ns	ns	0.035
AAT—Naming	ns	0.043	ns	0.018
AAT—Comprehension	ns	0.043	ns	0.018

*Significant after Bonferroni correction.

### Patients with M-NCD

Global cognition improved significantly, as indicated by the MODA and MMSE, in the whole sample as well as in both subgroups with and without AD. The whole sample and the group with M-NCD due to AD showed a statistically significant improvement also at the MoCA. When analyzing the sub-sessions of MODA, namely Orientations and Neuropsychological Tests, it must be noticed that all the groups improved in neuropsychological performance (M-NCD total sample: *p* = 0.017; M-NCD due to AD: *p* = 0.018; M-NCD non-AD: *p* = 0.025), whereas Orientations turned out to be improved in non-AD M-NCD only (*p* = 0.008).

No difference was found between pre- and post-treatment comparisons for reasoning ability, frontal functions and memory tests, except for the non-verbal Enhanced Cued Recall, in which a statistically significant difference was found in the whole sample (with a small Cohen’s *d*) and in the M-NCD due to AD. Selective attention improved in the whole sample, whereas in the two sub-groups with and without AD changes did not reach statistical significance.

Statistically significant differences were found for language abilities in the whole sample; namely, for syntactic comprehension of sentences (Token test; with a small Cohen’s *d*) and verbal repetition (but small Cohen’s *d*); in the sub-group with M-NCD due to AD, for syntactic comprehension, ecological comprehension (with a small Cohen’s *d*) and naming; and in the sub-group with M-NCD non-AD, for verbal repetition only.

Enhanced ideomotor praxis (Imitating Gestures test) was found in all the groups, whereas constructional praxis (Rey’s Complex figure-copy) reached a statistical significance in the whole sample and in the M-NCD due to AD.

In summary, following the g-ICA treatment, patients with M-NCD, both the whole sample and the two sub-groups with and without AD, showed improvements in global cognitive function and ideomotor praxis. The sub-group with M-NCD due to AD showed improvements also in some verbal language domains, such as verbal comprehension and naming, in constructional praxis and delayed visual memory. The sub-group with M-NCD non-AD showed improvements in verbal repetition and orientations. Reasoning abilities, verbal memory and executive functions did not appear to be susceptible of improvements.

### Patients with m-NCD

Patients with m-NCD showed statistically significant improvements in global cognitive functioning, reasoning, executive function, short term memory (digit span), delayed non-verbal memory (Rey’s complex figure-memory reproduction), selective attention, ideomotor praxis, and in all language domains (namely, syntactic and ecological verbal comprehension of sentences, naming, written language), except for repetition.

### Comparison between M-NCD and CG

The comparison between M-NCD and CG basically confirmed the results obtained from the comparison between the pre- and post-treatment assessments in patients with M-NCD (Table 4).

**Table 4:** **Results obtained from the comparison between M-NCD (*n* = 16) and CG (*n* = 11; Mann Whitney U test)**.

	M-NCD Δ means (±SD)	CG Δ means (±SD)	M-NCD vs. CG *p*
**Global cognitive function**
MODA	7.07 (4.9)	3.89 (4)	<0.001
MMSE	3.25 (3.24)	−2 (2.61)	<0.001
**Reasoning and cognitive level**			
Colored progressive
matrices	3.81 (6.72)	−2.36 (1.91)	0.004
**Memory**			
Digit span	−0.31 (0.95)	−0.27 (0.47)	ns
Corsi’s test	−0.06 (0.85)	−0.09 (0.3)	ns
Serial repetition of
two-syllabic words	0.19 (0.54)	0 (0)	ns
Rey’s 15 words-
immediate recall	0.13 (5.78)	−3.91 (2.91)	0.016
Rey’s 15 words-
delayed recall	0.31 (2.6)	−0.36 (0.92)	ns
Rey’s complex figure-
memory reproduction	1.22 (6.40)	−0.55 (1.97)	ns
Enhanced cued recall	1.00 (1.79)	−1.00 (1.1)	<0.001
**Selective attention**
Digit cancellation test	3.93 (8.46)	−2.18 (3.76)	0.008
**Praxis**
Imitating gestures
test	8.17 (8.01)	−5.59 (3.85)	<0.001
Rey’s complex
figure-copy	5.11 (6.43)	−1.91 (3.14)	0.005
**Frontal functions**
Frontal assessment
battery	1.20 (2.70)	−0.91 (1.04)	0.015
**Language**			
Aachener aphasia test
(AAT)—Token test	2.38 (2.96)	−1.64 (2.54)	0.001
AAT—Repetition	1.75 (2.11)	−1.18 (0.98)	<0.001
AAT—Written language	1.56 (3.14)	−0.64 (0.81)	0.014
AAT—Naming	3.88 (5.67)	−1.09 (0.70)	0.002
AAT—Comprehension	2.06 (6.88)	−0.73 (2.53)	ns

In fact, significant differences were found in the general cognitive functioning, in visual memory, selective attention, praxis, and in some language areas (syntactic verbal comprehension and repetition). Significant differences were obtained also in the visual-spatial reasoning (CPM), immediate verbal recall, frontal functions, naming, and written language.

## Discussion

The g-ICA is a short (6 weeks) combined intensive intervention based on mediation pedagogy and patient-centered principles. It is characterized by CS activities and cognitive tasks. Other multi-component approaches have been described in the literature (Avila et al., 2004; Olazarán et al., 2004, 2010; Sitzer et al., 2006; Raggi et al., 2007), usually implemented in patients with dementia. In our study, the combined treatment has also been implemented with m-NCD, due to the program flexibility and to customable tasks; furthermore, unlike other studies in the literature, in which results are usually described for AD or for patients with unspecified types of dementia (for a review see Alves et al., 2013), in our study results were also analyzed in the two sub-groups, with and without AD.

The whole M-NCD showed statistically significant improvements in almost all the investigated domains after treatment and most of these improvements were significantly higher than the changes observed in the CG in which worsening was the rule. As far as the global cognition is concerned, improvements were evident for the whole group with M-NCD and for both sub-groups (with and without AD); these positive modifications were found in all the global cognitive functioning batteries used, namely MoCA, MMSE and MODA, although *p* values were greater for MODA and MMSE than for MoCA: differences between *p* values might be ascribed to the fact that MoCA trials are more difficult than those included in the other two cognitive batteries, since MoCA enables to detect also a mild cognitive decline. These results are congruent with those from the literature, with regard to CS (Sitzer et al., 2006; Woods et al., 2012; Aguirre et al., 2013; Alves et al., 2013) and CT programs (Cipriani et al., 2006; Sitzer et al., 2006; Talassi et al., 2007; Alves et al., 2013), as well as to integrated programs (Olazarán et al., 2004). Our study also provides additional information about the global cognitive gains in patients with non-AD M-NCD. Moreover, the improved global cognition, indeed, seems to be more related to improvement in neuropsychological functioning and less to orientation for which, however, only patients with non-AD M-NCD showed a statistically significant improvement. These data might be explained by to the fact that orientation requires the use of memory, the cognitive function which first deteriorates in AD, in conjunction with attention and executive functioning (Traykov et al., 2007), and appears less susceptible of improvements following CS or training. In addition, from our study information about reasoning abilities and IQ—which were not improved—can also be derived: therefore, it can be hypothesized that improvements in global cognitive functioning are not related to increased IQ or reasoning abilities, but rather to more efficient cognitive functions as a whole. However, the comparison with the CG has shown a significant difference because the latter had a clear worsening; this might indicate that cognitive activation, even if was not able to improve the IQ, at least was able to maintain it at the pre-activation level; this was not true for the CG in which no activation was carried out.

Statistically significant results were obtained in Ideomotor praxis for all patients with M-NCD; this cognitive function has not usually been taken into account in the literature, despite the important role of motor components in the basic or instrumental daily living activities. Also for constructional praxis we found improved performance in the whole sample with M-NCD and in the sub-group with AD. Only marginal significance was found for changes in selective attention in the whole sample with M-NCD only; we believe that this result might be explained by the low power of our analysis due to its small sample size (a frequent methodological problem in rehabilitation studies) and this holds true for all the analyses we carried out.

In line with the results reported by the majority of studies in the literature (Alves et al., 2013; Choi and Twamley, 2013), no improvements in memory were found in our study, except for visual memory (Enhanced Cued Recall), in which a statistically significant result was obtained both for M-NCD as a whole (but with a small effect size) and M-NCD due to AD. This result is likely to be due to test administration and scoring, including induction of semantic processing, immediate cued recall four-by-four items, delayed free recall and delayed cued recall of all items; total scores included both free and cued recall (Grober et al., 1988), whereas, in the other memory tests, scores commonly include free recall only. As a whole, memory functions was very little improved; this result has an explanation in the early and severe memory deterioration in M-NCD, especially due to AD. The same applies for executive functions, in which no improvement was found. Nevertheless, the comparison between M-NCD and CG showed a statistically significant difference both in immediate verbal recall and in frontal functions: this result suggests that although g-ICA treatment did not cause improvements in these domains, it could be effective in slowing down their worsening.

Regarding language abilities, comprehension and repetition appeared to have improved in the whole sample, but effect sizes were small; this result in persons with M-NCD is very important, given the relevant role of language in communication and in mediating interpersonal relationships. The g-ICA, indeed, in addition to specific language tasks, includes a massive and continuous exposure to verbal language, since it is the principal vehicle of relationships between the patients and between patients and the mediator. To date, there is no study in the literature showing specific benefits of cognitive interventions on verbal language. Also in this case, the comparison with the CG group was able to disclose a slowing of the expected worsening of these functions.

Regarding m-NCD, to the best of our knowledge, our study is the only one in which an integrated cognitive approach has been applied. The review by Wang et al. (2014) has confirmed that cognition–based intervention (namely CT, CS, or memory training) can be effective on global cognitive functioning and less evidently, executive functions and delayed memory in patients with MCI; we also found improved global cognitive function and reasoning abilities (CPM scores). MMSE did not change but this battery is probably not appropriate for detecting a mild cognitive decline; in fact, all the patients in this group showed scores higher than 24 at the pre-treatment assessment.

Also in m-NCD, memory domains, especially verbal memory, were very little modified and some benefits were observed only in verbal span (serial repetition of two-syllable words) and delayed visual-spatial memory. In summary, the benefits obtained from g-ICA treatment on global cognitive and frontal functions in individuals with m-NCD confirm those reported earlier in the literature; however, we detected additional benefits on praxis ability, attention and verbal language.

This study has limitations and strengths. One limitation is the absence of a CG for m-NCD patients. A second limitation is the relatively small sample size of our groups, already cited above. Another limitation of this study is that a specific relation between one strategy and its effects cannot be established with certainty because the g-ICA is a package of combined strategies; a cross-over design (treating EG patients with all of the three techniques) would have been appropriate and would have probably provided more detailed data, but it was not possible to implement it because of a series of practical and regulatory limitations that do not allow us to follow patients intensively for a period of time long enough. Another important limitation of this program is the absence of labs specifically equipped for daily living activities, which directly address one’s own self-efficacy perception and therefore are fundamental, especially for persons with M-NCD. g-ICA includes procedures aimed to facilitate not only success in cognitive tasks, but also the general well-being of patients, but in this first study, which had the main objective to evaluate the cognitive effects of treatment, no measures of self-esteem, quality of life and self-awareness were used. Finally, this study had a baseline-treatment design that cannot allow strong conclusions on the direct cause of the effects detected, because of the impossibility to exclude competing hypotheses. However, our results are promising and indicate the need to carry out longer follow-up protocols in which an adequate CG can be included.

Despite the limitations, mediation pedagogy and the errorless learning can be considered to be important strengths of this study because they facilitate enhancement of motivation and removal of personal psychological barriers to cognitive treatment. Moreover, the rich neuropsychological battery has enabled the assessment of all the main cognitive functions, offering the opportunity to add useful information to the data already present in the literature. Since g-ICA is an intensive treatment, it allows patients to activate themselves cognitively for several hours, 5 days a week; on the contrary, previous studies in the literature usually describe treatments lasting for 45–50 min, delivered 4 days a week at most (Spector et al., 2003; Cipriani et al., 2006; Raggi et al., 2007; Talassi et al., 2007; Yamagami et al., 2012). The intensity of such a treatment was, most likely, another determining factor for the positive results obtained. The g-ICA turned out to be effective and well accepted by patients younger than 70–80 years, whereas studies in the literature have often reported results in older patients (Spector et al., 2003; Cipriani et al., 2006; Yamagami et al., 2012; Aguirre et al., 2013). Our treatment can be easily implemented in a hospital setting, it is cost-effective and represents an enrichment of the routine rehabilitation programs, as defined by the local regulations (Italian Government Essential Assistance Levels).

## Author Contributions

SP conceived the study. SP, DT, SM and RF contributed to the study design. SM, GP and CB participated in the diagnostic process and recruited the patients. DT, SM, FR and VC performed testing and data collection. DT and SM delivered the cognitive activation sessions. SP, AR and RF analyzed and interpreted the data. SP wrote the first version of the article. AR and RF provided critical revisions of the manuscript. All the authors read and approved the final version of the article.

## Conflict of Interest Statement

The authors declare that the research was conducted in the absence of any commercial or financial relationships that could be construed as a potential conflict of interest.
